# **Organoleptic, physicochemical, phytochemical and pharmacological evaluation of six medicated ghee used for** A**yurvedic management of Epilepsy**

**DOI:** 10.1016/j.jaim.2024.100995

**Published:** 2024-12-06

**Authors:** Snehal Moon, Nishikant Raut, Harshal Moon, Anmol Dhawande, Shailendra Gurav

**Affiliations:** a Department of Pharmaceutical Sciences, Rashtrasant Tukadoji Maharaj Nagpur University, Nagpur, Maharashtra, 440 033, India; b Department of Pharmacognosy, Goa College of Pharmacy, Panaji, Goa University, Goa, 403 001, India

**Keywords:** *Cow-ghee*, *Snehapaka*, *Medhya rasayana*, *Polyherbalformulation*, *Ayurvedicpreparation*, *Apasmara*, *Epilepsy*

## Abstract

**Background:**

Ayurvedic formulations need to be explored and tested with biomedical techniques. Polyherbal medicated *ghee* (*Ghrita*) are recommended for the management of epilepsy (*Apasmara*) and prepared using specialized process (*Snehapaka*), as per classical textbooks of Ayurveda. So, the present study deals with the systematic examination of the effect of different marketed formulations for treating *Apasmara* on convulsive impairment in Pentylenetetrazol (PTZ) induced seizures in mice.

**Objective:**

To assess organoleptic, physicochemical, phytochemical and pharmacological activity of selected *Ghrita* formulations used to treat *Apasmara.*

**Materials and Method:**

Six marketed *Ghrita* formulations used for *Apasmara,* such as Baidyanath *Brahmi Ghrita* (BBG), Patanjali *Brahmi Ghrita*, Kotakkal *Brahmi Ghrita*, *Panchagavya Ghrita*, *Mahapanchagavya Ghrita* and *Nirgundyadi Ghrita* were selected for the study. Selected *Ghrita* formulations were subjected to physicochemical analysis (following pharmacopeial procedures), phytochemical screening and pharmacological profile for quality and therapeutic efficacy. The screening parameters included pH, viscosity, specific gravity, loss on drying, acid value, saponification value, peroxide value, iodine value, refractive index and rancidity determination, and other phytochemical tests for secondary metabolites.

**Results:**

BBG demonstrates superior protection against the onset and duration of convulsions compared to alternative *Ghrita* formulations. As evidenced by its efficacy in mitigating PTZ-induced convulsions, BBG stands out as the optimal choice for exerting potent anticonvulsant effects.

**Conclusion:**

*In-vivo* screening suggests BBG as a potential *Ghrita* preparation for treatment of epilepsy.

## Highlights

• The medicinal efficacy of plant components is known to be increased when processed with cow *ghee.*
• In *Ayurveda*, polyherbal *Ghrita* is recommended for the management of *Apasmara.*
• The proposed work standardizes *Ghrita* formulations used mainly for treating *Apasmara.*
• *In-vivo* studies using PTZ-induced seizure models showcased the promising antiepileptic potential of *ghrita* formulations.

## 1 Introduction

**Fig. 1:**
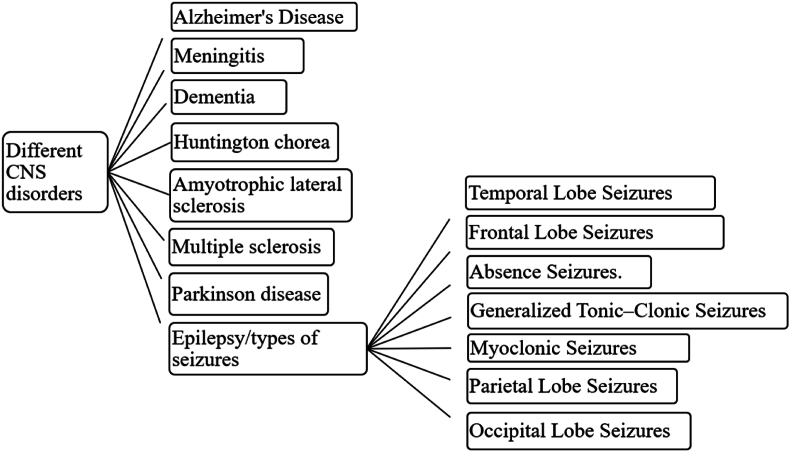
Various CNS disorders and types of epilepsy.

Epilepsy (*Apasmara*) is described as one of the eight life threatening diseases in Ayurveda classics. However, these diseases are challenging to treat and can be controlled to some extent with Ayurvedic remedies. Yet, sometimes, they remained uninhibited or uncured [1]. In modern perspectives, *Apasmara* is termed as epilepsy, a neurological disorder with abnormal brain activity causing convulsions or recurrent episodes of eccentric behaviour, perception and loss of recognition [2]. This neurological disorder, along with losing body tendency, develops in any individual irrespective of gender, age and race. The major social and economic implications of epilepsy include premature death, loss of self-confidence, regular health care, and loss of work productivity and attention. The antiepileptic treatment should ideally suppress the seizures without causing adverse effects. However, the treatment with antiepileptic drugs is associated with frequent severe adverse effects [3,4] (see Fig. 1).

*Ayurveda* provides concrete solutions to several acute and chronic diseases and disorders with minimal or no adverse effects, such as cost-effectiveness and improved patient compliance [5]. Incorporating cow *ghee* into Ayurvedic blends extends many health benefits, leveraging its nutritional richness. Their advantages are multifaceted, from purifying the blood to modulating the immune response, enhancing cognitive functions, optimizing digestion, and safeguarding against cardiovascular disorders. Cow *ghee* emerges as a potent therapeutic agent in addressing a spectrum of ailments, including ocular disorders, promoting wound healing, alleviating asthma, inflammation, cancer, neurological disorders, and various dermatological conditions. This therapeutic efficacy can be attributed to its composition rich in omega-3 and -9 fatty acids, essential vitamins A, D, E, and K, and short-chain fatty acids [6].

**Fig. 2:**
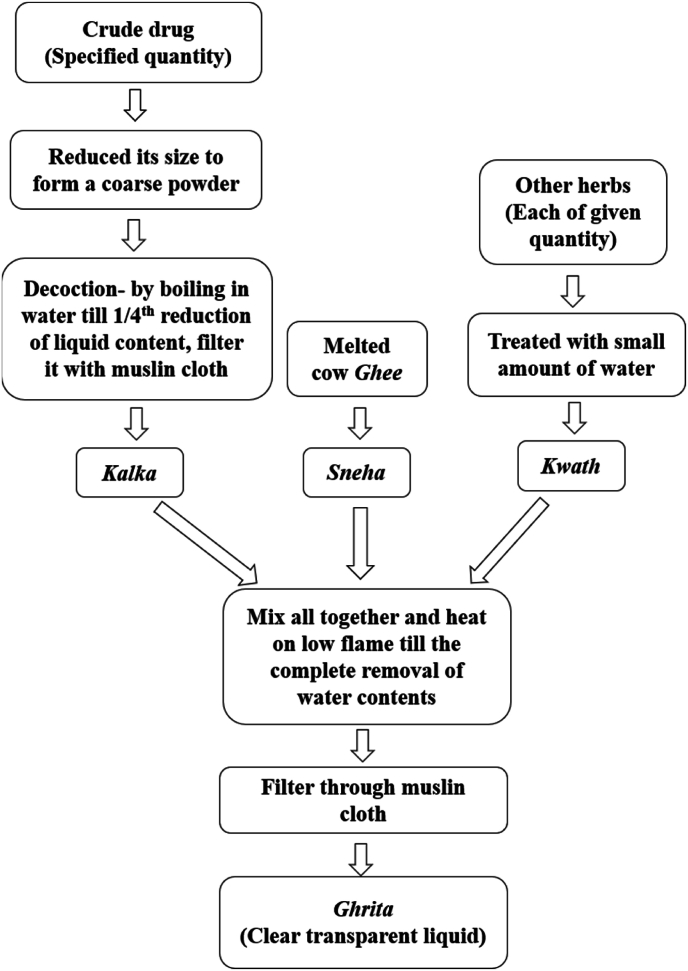
Method of preparation of *Ghrita* [10].

Consequently, cow *ghee* is a pivotal component, either as a standalone remedy or as an integral constituent in formulating medicated *ghee* preparations like *Ghrita*. Classical literature on *Ayurveda* reports more than 112 documented formulations for managing *Apasmara*, mainly including *Ghrita* exhibiting specific biological activities such as antiepileptic, antipsychotic, antidepressant and cognition-enhancing activity. *Ghrita* are *ghee*-based formulations mainly containing lipids and are commonly prescribed to treat various Central Nervous System (CNS) disorders, according to *Ayurveda*. For the formulation of *Ghrita*, cow *ghee* is processed with herbs to enhance and potentiate their activity to multifold [[6], [7], [8], [9]]. The standard method of preparation of *Ghrita* formulation following traditionally described procedures has been depicted in Fig. 2. The *Ghrita* is specially developed to target the delivery of medicaments and increase the bioavailability and therapeutic efficacy of herbal composition.

Though several formulations are documented for managing *Apasmara,* the actual number of *Ghrita* formulations that are especially available and in practice must be investigated [[11], [12], [13], [14]]. Accordingly, a market survey for the availability of *Ghrita* formulations was carried out which revealed that only six marketed *Ghrita* formulations such as Baidyanath *Brahmi Ghrita* (BBG), Patanjali *Brahmi Ghrita* (PBG), Kotakkal *Brahmi Ghrita* (KBG), *Panchagavya Ghrita* (PG), *Mahapanchagavya Ghrita* (MPG) and *Nirgundyadi Ghrita* (NG) are available having different compositions. Therefore, it was imperative to characterise and study the efficacy of these products. Consequently, the study was designed to characterise them for physicochemical parameters along with the therapeutic efficacy for the management of *Apasmara* employing pentylenetetrazol (PTZ) induced seizures in mice.

## 2 Materials and methods

### 2.1 Procurement of materials

Analytical grade solvents were used unless otherwise specified and used without further purification. PTZ was purchased from SIGMA-ALDRICH, Co., 3050 Spruce Street, St. Louis, MO 63103 USA 314-771-5765 SIGMA-ALDRICH CHEMIE GmbH, Riedstr. 2 D-89555 Steinheim 49 7329 970. All *Ghrita* preparations were procured from the retail pharmacy outlets. The details of *ghee*, Lamotrigine (LMT), and *Ghrita*, along with their sources, are given in Table 1.

**Table 1:** Details of *Ghrita* formulations.

Sr. No.	Name of formulation		Manufacturers	Batch No.	Mfg. Date	Exp. Date
1	BG	BBG	*Shree Baidyanath, Ayurved Bhavan* PVT. LTD. Gwalior Road, Jhansi, India	54/210	June/2019	May/2021
		PBG	*Divya Patanjali* Pharmacy, Lokmat square, Nagpur, India	BBRG 001	May/2019	Apr/2021
		KBG	*Arya Vaidya Shala*, Kottakkal, Kerala, India	BG/K57/19	Aug/2019	Jul/2022
2	PG		*Go-vigyan Anusandhan Kendra*, Deolapar, Nagpur, India	0301/19	May/2019	Apr/2021
3	MPG		*Arya Vaidya Shala*, Kottakkal, Kerala, India	MPG/23/19	Sep/2019	Aug/2022
4	NG		*Arya Vaidya Shala*, Kottakkal, Kerala, India	GN/40/19	Jul/2019	Jun/2022
5	Cow *ghee*		*Go-vigyan Anusandhan Kendra*, Deolapar, Nagpur, India	0185/19	May/2019	–
6	LMT		Zim Laboratories, MIDC, Kalmeshwar, Nagpur, India	19LM0003	Apr/2021	Mar/2024

*Brahmi Ghrita* (BBG), Patanjali *Brahmi Ghrita* (PBG), Kotakkal *Brahmi Ghrita* (KBG), *Panchagavya Ghrita* (PG), *Mahapanchagavya Ghrita* (MPG) and *Nirgundyadi Ghrita* (NG), Lamotrigine (LMT).

### 2.2 Organoleptic study

The suitability of *ghee*-based formulations depends upon their perceptual (organoleptic) characteristics, such as colour, taste, odour, texture, and touch, which are indications of rancidity. All the formulations are tested and observed at room temperature [10,15,16].

### 2.3 Physicochemical evaluation

Physicochemical estimation of all *Ghrita* samples was carried out as per the reported literature and standard Pharmacopeial procedures [10,17]. The experimental methods for the physicochemical assessments, such as pH, viscosity, specific gravity (SG), loss on drying (LOD), acid value (AV), saponification value (SV), peroxide value (PV), iodine value (IV), refractive index (RI) are given in the supplementary file [[16], [17], [18]].

### 2.4 Phytochemical screening

Phytochemical screening of all *Ghrita* samples was carried out as per the reported literature [10,17] and procedures mentioned in standard Pharmacopoeia. The experimental methods for the phytochemical screening, such as carbohydrates, proteins, alkaloids, phenols, steroids, and flavonoids, are in the supplementary file [19].

### 2.5 In-vivo animal studies

In this study, animals were utilized to examine the damage caused to the brain by exogenous epileptic agents to understand its physiological importance.

#### 2.5.1 Experimental design

This study was done in the laboratory of the Department of Pharmaceutical Sciences, Rashtrasant Tukadoji Maharaj Nagpur University, Nagpur, India, with strict observance of ethical guidelines and norms of the Committee for the Purpose of Control and Supervision of Experiments on Animals (CPCSEA). The Institutional Animal Ethics Committee (IAEC) of the mentioned institute approved the experimental protocol vide approval number IAEC/UDPS/2021/13 dated November 27, 2021. Swiss albino mice were issued from animal house.

The mice were of either sex and weighed about 20–30 g. Animals were issued and housed in a room in a 12 h light-dark cycle at 25°C ± 1 °C temperature along with relative humidity of 50–60%. Before one week of the experiment, animals were kept for acclimatization with free access to water and diet. Before 1 h of the experiment, animals were transferred to the laboratory, and protocols were followed during the day (08:00–18:00 h) [1].

#### 2.5.2 Animal groups

Mice were divided into nine groups of six animals each and treated with test drugs, as mentioned in Table 2. A fresh solution of LMT was made every day during experimentation. The oral gavage was used to administer samples up to a volume not more than 1 mL/100 g body weight. The doses of samples were decided from research articles, and the protocol duration was determined to be seven days. However, Ayurvedic principles stated that the efficacy of medicated *ghee* might be realized in 7 to 20 days [1,20].

**Table 2:** Details of the experimental protocol.

Sr. No.	Group code	Sample	Dose	Reference
1	Group I	Control group (normal saline)	10 mL/kg, p. o.	[21]
2	Group II	*Ghee*	5 g/kg p. o.	
3	Group III	Standard drug LMT	15 mg/kg p. o.	[20]
4	Group IV	BBG	300 mg/kg p. o.	[1]
5	Group V	PBG	300 mg/kg p. o.	
6	Group VI	KBG	300 mg/kg p. o.	
7	Group VII	PG	4 g/kg p. o.	[6]
8	Group VIII	MPG	1 g/kg p. o.	[22]
9	Group IX	NG	1 g/kg p. o.	

*Brahmi Ghrita* (BBG), Patanjali *Brahmi Ghrita* (PBG), Kotakkal *Brahmi Ghrita* (KBG), *Panchagavya Ghrita* (PG), *Mahapanchagavya Ghrita* (MPG) and *Nirgundyadi Ghrita* (NG), Lamotrigine (LMT).

The dosage selection for all formulations adhered to specific product literature guidelines. The control group received a standard oral dose of 10 mL/kg of normal saline solution [23]. In the case of *Brahmi Ghrita*, the study encompassed three consecutive doses: 100, 300, and 500 mg/kg, with the 300 mg/kg dose demonstrating significant results in convulsion management. Consequently, *Brahmi Ghrita* from three different manufacturers was administered at the effective dosage of 300 mg/kg [1]. Pre-treatment investigations with PG at a dosage of 4 gm/kg demonstrated complete protection against generalized tonic-clonic seizures alongside cognitive function enhancement [24]. Dosage determination for MPG and NG involved converting doses between animal and human subjects [22].

#### 2.5.3 Induction of seizures

On the 7th day of the experiment, LMT was given before 30 min of PTZ induction, and *Ghrita*s were administered before 1 h of PTZ treatment. A fresh PTZ solution was prepared in normal saline. Afterwards, each group were treated with 60 mg/kg of convulsive PTZ intraperitoneally after specified intervals, i.e., 1 h and 30 min for Group III. The onset and duration of different types of convulsions were observed. The doses were referred from various research articles and assimilated in our laboratory to prevent mortality. The suspension of myoclonic jerks and frequency of generalized tonic-clonic seizures (GTCS) drop of righting reflex were considered. The groups were ascertained for 30 min after the PTZ treatment [1].

#### 2.5.4 Statistical analysis

The statistical results of all groups in the form of mean ± SEM were analysed using one-way analysis of variance (ANOVA). The P value was considered noteworthy as it is less than 0.05. The free version of the GraphPad Prism and software performed all the statistical perusal.

## 3 Results

### 3.1 Organoleptic assessment of ghee, LMT and ghrita

Specific sensory properties of *ghee*, drug LMT and *Ghrita*s are depicted in Table 2. Varied perceptual characteristics (colour, odour and taste) of *ghee* were observed compared to the different *Ghrita* formulations. *Ghee* and other *Ghrita*s were similar in touch and texture, i.e., smooth, soft and greasy. The colour, odour and appearance of synthetic drug LMT are also included in Table 3.

**Table 3:** Organoleptic assessment of *Ghee*, LMT and *Ghrita*.

Sr No.	Name of formulation	Colour	Odour	Taste	Touch	Appearance
1	*Ghee*	Golden yellow	Buttery	Characteristic	Unctuous	Viscous, semisolid
2	BBG	Light green	*Ghee* like	Bitter	Unctuous	Viscous, semisolid
3	PBG	Slightly yellowish	*Ghee* like	Bitter	Unctuous	Viscous, semisolid
4	KBG	Green	*Ghee* like	Bitter	Unctuous	Viscous, semisolid
5	PG	Green	*Ghee* like	Bitter	Unctuous	Viscous, semisolid
6	MPG	Green	*Ghee* like	Bitter	Unctuous	Viscous, semisolid
7	NG	Yellow orange	Pungent, *Ghee* like	Bitter	Unctuous	Viscous, semisolid
8	LMT	White	Characteristic	–	Amorphous	Powder

*Brahmi Ghrita* (BBG), Patanjali *Brahmi Ghrita* (PBG), Kotakkal *Brahmi Ghrita* (KBG), *Panchagavya Ghrita* (PG), *Mahapanchagavya Ghrita* (MPG) and *Nirgundyadi Ghrita* (NG), Lamotrigine (LMT).

### 3.2 Phytochemical screening

The qualitative analysis, i.e. *Ghrita's* phytochemical screening clearly revealed the presence of different metabolites, such as carbohydrates in BBG and MPG; proteins and amino acids in PG, MPG and NG; alkaloids in PG, MPG and NG; steroids in *Ghee*, BBG, PBG, KBG, PG and NG whereas phenolics (and tannins) and flavonoids were found in all test samples (Table 4).

**Table 4:** Results of phytochemical screening of *Ghrita*.

Sr. No.	**Metabolites**	←Samples→						
		*Ghee*	BBG	PBG	KBG	PG	MPG	NG
1	Carbohydrate	–	+	–	–	–	+	–
2	Protein & amino acid	–	–	–	–	+	+	+
3	Alkaloids	–	+	+	+	–	+	+
4	Phenolics	+	+	+	+	+	+	+
5	Steroids	+	+	+	+	+	–	+
6	Flavonoids	+	+	+	+	+	+	+

+present − absent.

*Brahmi Ghrita* (BBG), Patanjali *Brahmi Ghrita* (PBG), Kotakkal *Brahmi Ghrita* (KBG), *Panchagavya Ghrita* (PG), *Mahapanchagavya Ghrita* (MPG) and *Nirgundyadi Ghrita* (NG), Lamotrigine (LMT).

### 3.3 Physicochemical evaluation

The results of physicochemical characterization following standard procedures are detailed in Table 5.

**Table 5:** Physicochemical evaluation of cow *Ghee* and *Ghrita* formulations.

Sr. No.	Physicochemical Parameters ↓	←Samples→						
		*Ghee*	BBG	PBG	KBG	PG	MPG	NG
1	pH	6.1 ± 0.001	5.44 ± 0.01	5.19 ± 0.01	5.42 ± 0.01	6.52 ± 0.01	4.69 ± 0.01	4.86 ± 0.01
2	Viscosity (cP)	60218 ± 0.33	31822 ± 10.68	36302 ± 20.09	150134 ± 7.83	250175 ± 16.3	33484 ± 9.56	35867 ± 13.89
3	SG g/mL	0.8513 ± 0.0001	0.9015 ± 0.00	0.8979 ± 0.002	0.8925 ± 0.001	0.7223 ± 0.01	0.914 ± 0.01	0.905 ± 0.01
4	LOD %	7.421 ± 0.01	3.906 ± 0.01	8.6007 ± 0.01	11.130 ± 0.01	8.2317 ± 0.01	4.515 ± 0.01	8.493 ± 0.012
5	AV	2.066 ± 0.01	2.455 ± 0.02	3.254 ± 0.0005	4.636 ± 0.001	1.651 ± 0.01	5.663 ± 0.01	0.779 ± 0.01
6	SV	50.569 ± 0.03	91.951 ± 0.0002	32.619 ± 0.04	119.6 ± 0.03	36.160 ± 0.01	106.6 ± 0.01	101.624 ± 0.01
7	PV	67.319 ± 0.04	52.93 ± 0.002	195.72 ± 0.19	106.44 ± 0.03	61.3623 ± 0.01	134.3 ± 0.03	103.39 ± 0.02
8	IV	26.218 ± 0.01	21.965 ± 0.003	26.522 ± 0.01	16.170 ± 0.0012	21.5227 ± 0.01	24.8 ± 0.01031	20.4917 ± 0.01
9	RI	1.633 ± 0.01	1.655 ± 0.002	1.644 ± 0.001	1.636 ± 0.001	1.6393 ± 0.01	1.66 ± 0.01	1.65 ± 0.001

All experiments are performed in triplicate, and values are expressed as Mean ± SEM.

*Brahmi Ghrita* (BBG), Patanjali *Brahmi Ghrita* (PBG), Kotakkal *Brahmi Ghrita* (KBG), *Panchagavya Ghrita* (PG), *Mahapanchagavya Ghrita* (MPG) and *Nirgundyadi Ghrita* (NG), Lamotrigine (LMT).

### 3.4 In-vivo study

In mice pre-treated seaparately with control saline, standard drug LMT and six different marketed *Ghrita* formulations respectively, the nature and severity of the onset and duration of convulsions were observed. The results are given in Table 6 and Table 7.

**Table 6:** Effect of cow *ghee* and *Ghrita* samples against PTZ-induced convulsions.

Sr. No.	Drug treatment	Convulsion (seconds)			Status (or) no. of animals alive/no of animals used	% Protection of mortality
		Onset	Duration	Nature and severity		
**1**	**Control saline**	25 ± 0.2593	1273 ± 2.4864	Severe convulsion, dead-like	5/6	83%
**2**	***Ghee***	39.33 ± 0.4352	1197.33 ± 8.7096	Moderate convulsions	6/6	100%
**3**	**LMT**	20.17 ± 0.3179	886 ± 2.0234	Very mild convulsions	6/6	100%
**4**	**BBG**	31 ± 0.1315	179.5 ± 0.386	Mild Jerky movement and convulsion	6/6	100%
**5**	**PBG**	22.17 ± 0.1821	216.83 ± 0.4854	Jerky movement and convulsion	6/6	100%
**6**	**KBG**	20.67 ± 0.1019	326.17 ± 0.6642	Jerky movement, Straub tail and convulsion	5/6	83%
**7**	**PG**	31.5 ± 0.2896	272.17 ± 1.7789	Jerky movement and convulsion	5/6	83%
**8**	**MPG**	92.83 ± 1.2549	739.83 ± 2.5961	Jerky movement, Straub tail and convulsion	6/6	100%
**9**	**NG**	24.5 ± 0.2863	336.67 ± 1.3935	Jerky movement, Straub tail and convulsion	5/6	83%

*Brahmi Ghrita* (BBG), Patanjali *Brahmi Ghrita* (PBG), Kotakkal *Brahmi Ghrita* (KBG), *Panchagavya Ghrita* (PG), *Mahapanchagavya Ghrita* (MPG) and *Nirgundyadi Ghrita* (NG), Lamotrigine (LMT).

Numerals are manifested as Mean ± SEM (n = 6) p < 0.0001 (contrasted to a control group) by employing Ordinary One-way Analysis of Variance (ANOVA) followed by Bartlett's test.

**Table 7:** Details of ANOVA summary by post-hoc test analysis.

Sr. No.	Parameters		Onset of convulsions	Duration of convulsions
1	F value		10.10	30.88
2	P value		<0.0001	<0.0001
3	Significant diff. among means (P < 0.05)?		Yes	Yes
4	R square		0.6422	0.8459
5	Brown-Forsythe test	F (DFn, DFd)	3.179 (8, 45)	3.521 (8, 45)
6		P value	0.0061	0.0031
7		Are SDs significantly different (P < 0.05)?	Yes	Yes
8	Bartlett's test	Bartlett's statistic (corrected)	48.25	91.56
9		P value	<0.0001	<0.0001
10		Are SDs significantly different (P < 0.05)?	Yes	Yes
11	Treatment (between columns)	SS	25112	8919993
12		DF	8	8
13		MS	3139	1114999
14		F	(8, 45) = 10.10	(8, 45) = 30.88
15	Residual (within columns)	SS	13992	1624714
16		DF	45	45
17		MS	310.9	36105

## 4 Discussion

*Ghrita* is prepared by processing cow *ghee* with herbs that enhance and potentiate their activity to multifold. However, very few scientific evidences are available on it [6]. Also, the market survey indicates very few *Ghrita* formulations with different compositions for *Apasmara* are available. So, it was necessary to characterise these products based on various phytochemical, physicochemical, and pharmacological parameters.

The colour of the *Ghrita* was observed to be greenish due to the presence of individual contents like *Brahmi*, cow dung in PG, many herbal medicaments, etc., which were incorporated to achieve specific effects of formulation. Moreover, the base of the formulation was cow *ghee*. Hence, the odour was *ghee* -like, and the resultant formulations were viscous and sticky. The bitter/characteristic taste of the formulations was because of the addition of herbal constituents.

The benedict test indicated the presence of a trace amount of glucose in MPG and NG. The presence of amino acids was observed in the cow *ghee* samples. Alkaloids, nitrogenous compounds present in the formulation, are considered valuable medicinal constituents derived from plant sources due to their medicinal activity. The pH and SG were determined at 32°C. As SG increases, solid content is raised compared to liquid content, which enhances the shelf life and therapeutic value of BBG, MPG and NG [8,15].

LOD reveals the contiguity and quantity of moisture in the sample. Hence, BBG is an adequate and suitable quality product, as it has less LOD value than other samples. The acid value quantifies how many carboxylic acid groups are present in a sample, like a fatty acid. Triglycerides were transformed into fatty acids and glycerol as oil-fats began rancidifying, raising the acid value. A low acid value indicates less probability of degradation of *Ghrita*; ultimately, it improves its medicinal value and longevity [17]. According to the results, MPG had a higher acid value than other *Ghrita*s, indicating that *Ghrita* was hydrolysed during the *Snehapaka* process, which may be aided by the interaction of triglycerides with the active components in MPG, resulting in the formation of two by-products, one is glycerol, and other is free fatty acids. Lesser free fatty acid (less acid value) shows that the stability and shelf life of BBG were greater than those of MPG and NG.

The sample's relative molecular mass of fatty acids is calculated by saponification value, which is directly proportional to its fatty matter. The low saponification value in long-chain molecules compared to short-chain fat molecules is due to the lesser functional group, i.e., the carboxylic group per unit mass. Long-chain fatty acids take more time to absorb than short-chain acids. Meanwhile, medium-chain triglycerides are known for their easy metabolism in humans and are a biologically passive energy source. These are diffused quickly from the gastrointestinal tract without modifying their structure to the portal system [25]. Increased saponification value of BBG showed that it has greater short-chain and medium-chain fatty acids than other samples.

The peroxide value test is used to evaluate the oxidative rancidity of a substance. The quantity of peroxide oxygen in 1 kg of lipid is known as the peroxide value. The peroxide value of fat is a measure of rancidity during storage. The double bond present in fats plays a vital role in auto-oxidation. The leading test for oxidative rancidity is the determination of the peroxide value. Peroxides are recurrent products in the auto-oxidation process. Auto oxidation is an oxygen-based free radical process that results in the degradation of fats and oils, which create unpleasant smells and scents [26]. The lowest peroxide value of BBG indicated its reduced oxidative rancidity compared to other samples.

The amount of iodine in grams is engrossed by 100 g of a substance is measured by the Iodine value, also known as “Iodine adsorption value”, “Iodine number” or “Iodine index”. The critical application of iodine number is determining the amount of unsaturated content in fatty acids. Supplementing with unsaturated fats increases the total dietary energy consumption to the required amount while having no adverse effects on blood lipids [27]. The iodine value of all the samples of BBG, PBG was more than KBG and NG, denoting its high therapeutic value. The greater Iodine number enhances the nutritional status and therapeutic value of *Ghrita*, due to the presence of more unsaturated fatty acid linkage in formulation.

The refractive index is the proportion of the velocity of light in oil or fat to that of the velocity in a vacuum. It is a fundamental characteristic of a substance frequently used to identify a specific substance, verify its purity, or determine its concentration. The refractive index of all the samples of *Ghrita* revealed that few active components were present in the preparation of *Ghrita*s, and it was in the normal range. Also, the rancidity test was negative in all the samples.

For *in-vivo* studies, many rodent generalized seizure models, such as PTZ and BIC-induced models, are described in the literature [28]. They all show pharmacologic selectivity for the GABA-ergic action of antiepileptic drugs and exhibit behavioural and Electroencephalogram (EEG) similarity to human seizures. These models are characterised, predictable and reproducible. They help examine the mechanism underlying the aetiology of clonic seizures and test prospective for antiepileptic drugs for anticonvulsant activity and ayurvedic antiepileptic formulations [29]. As a model for generalized seizures, PTZ-induced seizures in mice were used in the study to demonstrate the antiepileptic efficacy of formulations. It is widely known that PTZ decreases the amount of Cl ions that enter the membrane and blocks GABA's specific binding to the GABA A-receptor Cl-ion channel complex on the membrane of neurons [30]. It is now commonly accepted that PTZ acts at the picrotoxin site of the GABA A receptors/Cl-ionophore complex [31]. When the receptor is activated, the Cl channel of the receptor opens, allowing an influx of Cl to enter and hyperpolarise the neuron [32].

In the realm of Ayurvedic medicine, *Ghrita* preparations are deeply rooted in traditional Indian medicine and offer promising therapeutics in the management of various CNS disorders due to their multifaceted pharmacological actions. These formulations are often enriched with medicinal herbs known for their neuroprotective and cognitive-enhancing properties. Research demonstrated the synergistic combination of herbs in these preparations exerts neurotrophic effects, modulates neurotransmitter levels, and attenuates oxidative stress, thereby conferring therapeutic benefits for a spectrum of CNS disorders, including anxiety, depression, and neurodegenerative diseases. Ancient Ayurvedic texts such as the *Ayurved Sarsangraha* and *Sushruta Samhita* provide foundational insights into the formulation and application of these remedies [13,33]. Contemporary scientific investigations, exemplified by the reported investigations [1,24,[34], [35], [36], [37], [38]], continue to elucidate the pharmacological mechanisms underpinning the efficacy of Ayurvedic *Ghrita* preparations in CNS disorders, effectively bridging ancient wisdom with modern scientific validation.

Earlier studies described the effects of *Ghrita* in the treatment of CNS disorders and it can be a choice of adjuvant drug to improve brain functions. The *Brahmi Ghrita* has potent anticonvulsive action that revitalises brain functions. It further repairs the oxidative damage caused by synthetic drugs [37]. The antioxidant activity is common in all the *Ghrita* formulations [10,15,17]. The observed attenuation of oxidative stress further corroborates the traditional claims of these preparations in ameliorating conditions such as anxiety, depression, and neurodegenerative diseases. These studies add to the growing body of evidence supporting the efficacy of Ayurvedic *Ghrita* preparations in CNS disorders, validating the insights provided by ancient Ayurvedic texts. The reported studies [36,37] investigated the therapeutic potential of *Panchagavya Ghrita* (PG), which is traditionally used for various ailments, including epilepsy, anxiety, fever, and jaundice. PG demonstrated antiepileptic effects in a maximal electroshock (MES) induced seizure model in rats, mitigating cognitive impairment and oxidative stress.

Additionally, when administered alongside sub-therapeutic doses of phenytoin (PHT) and carbamazepine (CBZ), PG exhibited enhanced antiepileptic effects without significant alteration in serum levels of PHT and CBZ, suggesting its potential as an adjunct therapy for epilepsy with improved efficacy and tolerability. The study on PG reported that its pretreatment at a dose of 4 g/kg gives 100% protection against generalized tonic-clonic seizures. It can be used as medicine for oxidative stress and improved cognitive activities in seizure protection. The polyherbal formulation of *Sarasvata Ghrita* improves intelligence and memory. It is also used to treat speech delay and speaking difficulties in children. It significantly elevates the dopamine, noradrenaline and 5-hydroxytryptamine levels in the brain, resulting in efficient neuroprotective and memory-enhancer activity [35]. Another study of *Kalyanaka ghrita, Panchagavya ghrita, Brahmi ghrita and Mahapanchagavya ghrita* has shown a significant reduction of mild to moderate depression [39].

In the present study, antiepileptic activity was assessed based on the results of the *in-vivo* study. It was predicted based on the onset of convulsions, the duration of convulsions, the nature and severity of convulsions, the number of animals alive after the experiment and the percent protection of mortality. The onset and duration of convulsions were recorded in seconds, and ±SEM was calculated. The results of all groups were demonstrated with the help of ordinary One-way Analysis of Variance (ANOVA), further by Bartlett's test. Subsequently, it indicates that the onset of convulsions was delayed in MPG, similar to other formulation treatments. However, the duration of convulsions was much less in BBG than in any other comparative formulations with 100% protection.

Future studies should delve into the comprehensive evaluation of these formulations, including their efficacy, safety profile, and potential clinical applications, to enrich our understanding and potentially integrate them into mainstream medical practices. Additionally, research endeavours could focus on refining the processing techniques and exploring novel approaches to optimize the therapeutic outcomes of *Ghrita* formulations, thereby catering to evolving healthcare needs.

## 5 Conclusion

Our research emphasizes the critical need to characterise *Ghrita* formulations, mainly for treating *Apasmara*, due to the limited availability of scientifically validated products. We revealed significant variations among formulations through comprehensive assessments encompassing phytochemical, physicochemical, and pharmacological parameters, highlighting the importance of quality control. The observed greenish hue and characteristic odour, along with the presence of amino acids and alkaloids, revealed these formulations' complexity and potential therapeutic efficacy. Furthermore, parameters such as specific gravity, % LOD, acid value, peroxide value and other parameters offer insights into the stability and shelf life of the formulations, with BBG demonstrating superior qualities. Notably, the *in-vivo* studies using PTZ-induced seizure models showcased BBG's promising antiepileptic potential, particularly in reducing convulsion onset and duration. These findings underscore the viability of characterised *Ghrita* formulations, especially BBG, as effective interventions for conditions like *Apasmara*, warranting further clinical investigations and validation.

## Author contributions

NR and SG: Conceptualization, Methodology, Validation, Investigation, Data curation, Writing – review & editing. SM,: Methodology, Investigation, Validation, Writing – original draft. HM, and AD: Formal analysis, Resources, Validation, Drafting of MS, Writing – review & editing.

## Data availability statement

Data will be made available on request.

## Source of funding

The research received grant from Dr. Babasaheb Ambedkar Research and Training Institute, Pune.

## Declaration of generative AI in scientific writing

NIL.

## Conflict of interest

The authors declare that they have no known competing financial interests or personal relationships that could have appeared to influence the work reported in this paper.

Dr Shailendra Gurav is also one of the co-authors of the present manuscript. Dr. Shailendra Gurav is a part of JAIM's editorial board and was not involved in any manuscript review or editorial processes.
