# Supplementary material for: Organoleptic, physicochemical, phytochemical and pharmacological evaluation of six medicated ghee used for Ayurvedic management of Epilepsy
Source: J Ayurveda Integr Med. 2024 Dec 6;15(6):100995. doi: 10.1016/j.jaim.2024.100995 (PMC11667154; doi:10.1016/j.jaim.2024.100995)
Supplement: Multimedia component 1 [file mmc1.doc]

**SUPPLEMENTARY FILE**

**COMPARATIVE SCREENING OF *GHRITA* FORMULATIONS USED FOR THE TREATMENT OF *APASMARA***

- - 1. **pH:**

The pH of all samples was determined with the help of a Mettler Toledo pH meter.

- - 1. **Viscosity:**

The viscosity of samples was determined by DV1 Digital Viscometer. The U tube was filled with samples, and the spindle L3 was attached to determine the viscosity on 100 rpm of tested samples (9, 10, 15-16).

- - 1. **Specific gravity:**

The gravity bottle was cleaned with detergent and rinsed with distilled water, acetone, and ether. The weight of the empty dried bottle was noted. Then, distilled water was filled in the bottle, covered with a lid, and the extra water was removed with tissue paper. Again, the weight of the bottle with water was noted. The same process was repeated for all samples of lipids (9, 10, 15-16).

- - 1. **Loss on Drying at 110°C:**

The dried crucible was weighed with about 5 g of sample, and the weight before and after adding sample was recorded. Then, the crucible was placed in an oven for about 2 h at 110°C. After that, the crucible was placed in the desiccator for 30 min, with the help of gloves or tongs. The crucible was weighed and repeated until the constant weight was attained (9, 10, 15-16).

The difference between actual weight and weight loss after heating was calculated, and this percentage was the % LOD at a certain temperature. The time of deterioration of the sample depends on the proportion of moisture in the sample. As moisture content increases, there are more chances of fungal growth and early deterioration of the sample (9, 10, 15-16).

- - 1. **Acid Value (AV):**

The sample of about 10 g was weighed and mixed with 50 mL of solvent (neutralised acid-free mixture of alcohol and ether each of 25 mL). Titrate the solution with 0.1N potassium hydroxide solution using about 1 ml of phenolphthalein indicator. The pale pink colour appears for 15 sec as the endpoint. The same process was repeated for each sample.

In the oxidation process, triglycerides get converted into glycerol and fatty acids responsible for the sample's acidity. Lipase is the lipolytic enzyme resulting from hydrolysis, and the thermal effect discharges fatty acids from the samples; hence, AV is directly proportional to rancidity. The AV of formulations in a range of 2 indicates better quality (9, 10, 15-16).

Acid Value = 56.1 x N x n

W

Where N = Normality of KOH

n = No. of ml of 0.1 N KOH required

W = weight of sample

- - 1. **Saponification value (SV):**

In a tared beaker, about 2 g of sample was weighed and mixed with 3 mL of 1:1 ratio ethanol/ether solution. Transfer the solution to a round bottom flask with three subsequent washing of 7 mL of each solvent. The flask was attached to the reflux condenser for heating in water bath for 30 min after adding 25 ml of 0.5 N alcoholic KOH into the solvent. Repeat the procedure for blank titration. Then, cool the flasks at room temperature for some time. Titrate the solution by 0.5 N HCl using phenolphthalein as an indicator. The reading of the endpoint was noted for all the samples (9, 10, 15-16).

The deviation in the reading of the sample and blank gives the required ml of 0. 5 N KOH to saponify 1 g of lipid/fat. The SV of fats shows mg of KOH absorbed by 1 g of fat.

Saponification value = (B - S) x 28.05

W

Where, B= ml of HCl for blank

S= ml of HCl for sample

W = weight of sample

- - 1. **Peroxide value (PV):**

About 5 g of sample was weighed and mixed in the conical flask with 30 mL of the mixture (3 volume of glacial acetic acid and 2 volume of chloroform). Then 0.5 mL of potassium iodide saturated solution was added and stood for 1-2 min (16). Then 30 mL of water was added before the titration, and with continuous shaking, the titration proceeded with the solution with 0.01 M sodium thiosulphate till the yellow colour vanished. After adding 0.5 mL of starch indicator, titration was continued till the withdrawal of blue colour. With the same method, blank reading was taken by dropping the sample (9, 10, 15-16).

Peroxide value = 10 (B- S)

W

Where B= ml of sodium thiosulphate for blank

S = ml of sodium thiosulphate for sample

W = weight in g of the sample.

- - 1. **Iodine value (IV):**

The ‘TEST’ solution was prepared by dissolving about 10 mL of sample with 10 mL of chloroform and 20 mL of iodine monochloride reagent in an iodine flask. Similarly, ‘BLANK’ solution was prepared by omitting the sample. Then, both the solutions were mixed and kept in a dark place for 30 min. After the incubation of solutions for 30 min, 10 ml of saturated solution of potassium iodide in water was added to each flask. The sides of the flask and stopper were rinsed with 50 mL of distilled water. Titrate these solutions with 0.1 M standardized sodium thiosulphate until a pale straw colour appears. Then, 1 mL of starch solution was added, and a purple colour solution was added. Then, the titration continued until the solution became colourless for recording the endpoint. The same procedure of titration was repeated for BLANK. The calculation was carried out using the following formula (9, 10, 15-16).

Iodine Value = 12.69 (B - S) N

W

Where, B = volume in ml of Sodium thiosulphate required for the blank

S = volume in ml of Sodium thiosulphate required for the sample

N= normality of the standard Sodium thiosulphate solution

W= weight in g of the sample.

- - 1. **Refractive index (RI):**

The RI of selected *Ghrita* formulations was measured using Abbe’s refractometer. Water was used to calibrate the apparatus as a liquid and specific monochromatic light source. The boundary between the bright and dark area was adjusted to focus by a micrometre screw; also, the telescope’s cross wire was fixed on the boundary at a refractometer scale uniting the bright-dark background. The procedure was repeated after the calibration of the equipment. The reading of RI was taken by putting a drop of water on the prism, and the drive knob controlled the boundary of intersects accurately at centre. The result was 1.3325, noted at 25^o^C. The variation in the reading of calibration and reading of water, i.e.. 1.3325 shows the error of the refractometer. The reading of the sample needs to be corrected in plus (+) if the error of reading is <1.3325 and vice versa for error in minus (-). Also, RI may differ with changes in temperature and wavelength. The fat structure includes different proportions of derivatives like fatty acids and esters, which ultimately change with temperature or other constituents or due to any impurities incorporated (9, 10, 15-16).

- - 1. **Rancidity:**

The sample of melted *Ghritas* of about 1 mL was treated with 1 mL con. HCl. To this solution, 1 % phloroglucinol in diethyl ether of about 1 mL was added. The slight oxidation of the fat sample would be observed by the pink colour of the resulting solution, whereas the red colour depicted the definite oxidation of the sample (9, 10, 15-16).

- 1. **Phytochemical screening: qualitative tests:**

The ethanolic extract of *Ghrita* formulations was examined for the presence of the phytochemical constituents with the help of standard analytical methods. Qualitative analysis detected different active constituents like carbohydrates, lipids, alkaloids, turpentines, proteins, etc. (9, 10, 15-16).

The sample of *Ghrita* was mixed with about 5 mL alcohol and filtered the solution; these filtrates were further used to analyse phytoconstituents.

**2.4.1 Carbohydrate:**

**a. Benedict test:**

The filtrate was taken in a test tube, and Benedict's reagent was added to it. Upon heating in a water bath, the orange-red precipitate shows the appearance of reducing sugar in a sample (9, 10, 15-16).

**b. Fehling’s test:**

Fehling’s solution A and Fehling’s solution B in equal proportions of about 1 mL were mixed with 2 mL of extract in the test tube. Upon heating for 1-2 min, brick red or red precipitate indicates carbohydrates in samples (9, 10, 15-16).

**2.4.2 Million’s Test for Protein and Amino Acids:**

The alcoholic extract and 2 mL million’s reagents were mixed in the test tube, giving a white precipitate. Upon heating the tested solution, the white precipitate turned red, showing the presence of protein (9, 10, 15-16).

**2.4.3 Alkaloids:**

The alkaloidal reagent was used along with the filtrate of extract of the sample and dilute HCl.

**Hager’s Test:** The filtrate of samples was treated with a picric acid solution (Hager’s reagent), and the presence of alkaloids can be identified by the formation of a yellow precipitate (9, 10, 15-16).

**2.4.4 Phenol & tannins:**

**Ferric Chloride Test:**

In the alcoholic extract of samples, a few drops of neutral ferric chloride solution were mixed to examine blue, green, purple-red or orange colour, indicating phenols & tannins (9, 10, 15-16).

**2.4.5 Steroids:**

**Salkowski Reaction:** In a test tube, 2 mL of chloroform extract of samples were taken and slowly, from the side of a test tube, 1 mL of conc. H_2_SO_4_ was added. Steroids can be identified by the reddish chloroform layer (9, 10, 15-16).

**2.4.6 Flavonoids:**

**Shinoda’s test:** In test-tube, 5-10 drops of dil. HCl were mixed with 0.5 mL of alcoholic extract of the samples accompanied by a small piece of magnesium. Existence of flavonoid in sample observed by pink, reddish pink or brown colour (9, 10, 15-16).
